# Supplementary material for: Prefrontal working memory signal primarily controls phase-coded information within extrastriate cortex
Source: bioRxiv. 2024 Aug 29:2024.08.28.610140. Preprint. [Version 1] doi: 10.1101/2024.08.28.610140 (PMC11383686; doi:10.1101/2024.08.28.610140)
Supplement: Supplementary file 1 [file media-1.pdf]

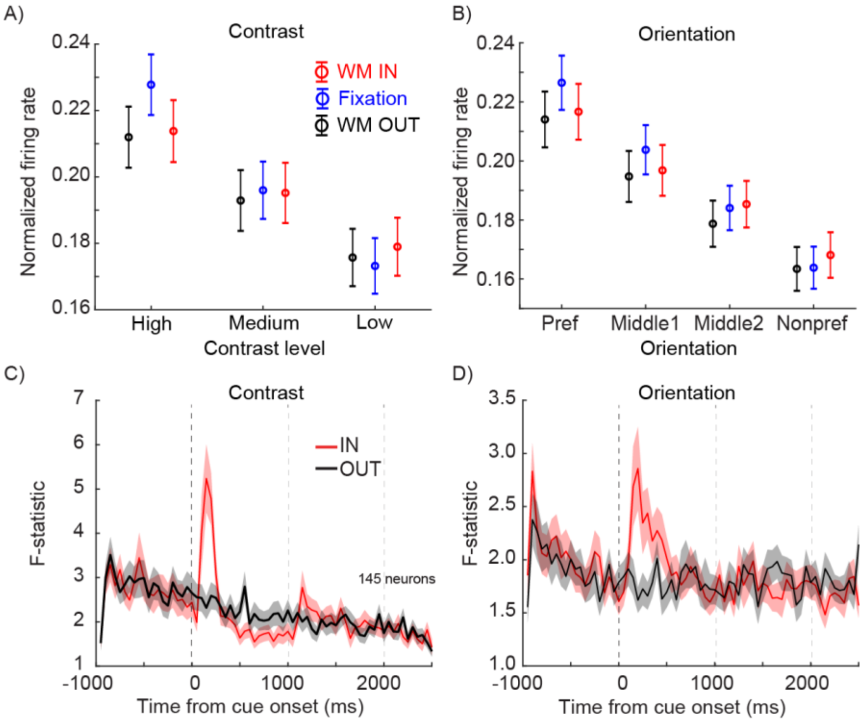

Figure S1) V4 neurons' sensitivity to stimulus contrast and orientation is not altered by WM. To determine whether the top-down WM signal interacted with the bottom-up sensory input to modulate V4 firing rates, we compared the firing rate of V4 neurons across different background stimuli and WM conditions. A, B) Mean normalized firing rate of 145 V4 neurons during fixation (blue), the delay period of working memory IN (red), and the delay period of working memory OUT (black) for different background stimulus contrasts (A) and orientations (B). Orientations were sorted by the neuron's preference, as measured by firing rate during the fixation period (Pref, highest firing rate; Nonpref, lowest firing rate). Error bars show standard error of the mean (SEM). C, D) Time course of averaged F-statistic values based on one-way ANOVA for discrimination between three different contrasts (C) or between four different orientations (D) for IN (red) and OUT (black). Sensory discrimination peaked just following cue onset during the IN condition. However, there was no significant difference in discriminability for different background stimuli between the IN and OUT conditions during the delay period, either for stimulus contrast (Contrast: $discriminability_{IN}$= 1.995±1.997, $discriminability_{OUT}$= 1.910±1.866, p=0.139) or for orientation (Orientation: $discriminability_{IN}$= 1.771±1.613, $discriminability_{OUT}$= 1.814±1.508, p=0.269). Thus, the top-down WM signal did not alter V4 neuron's firing rates in response to different bottom-up visual input.

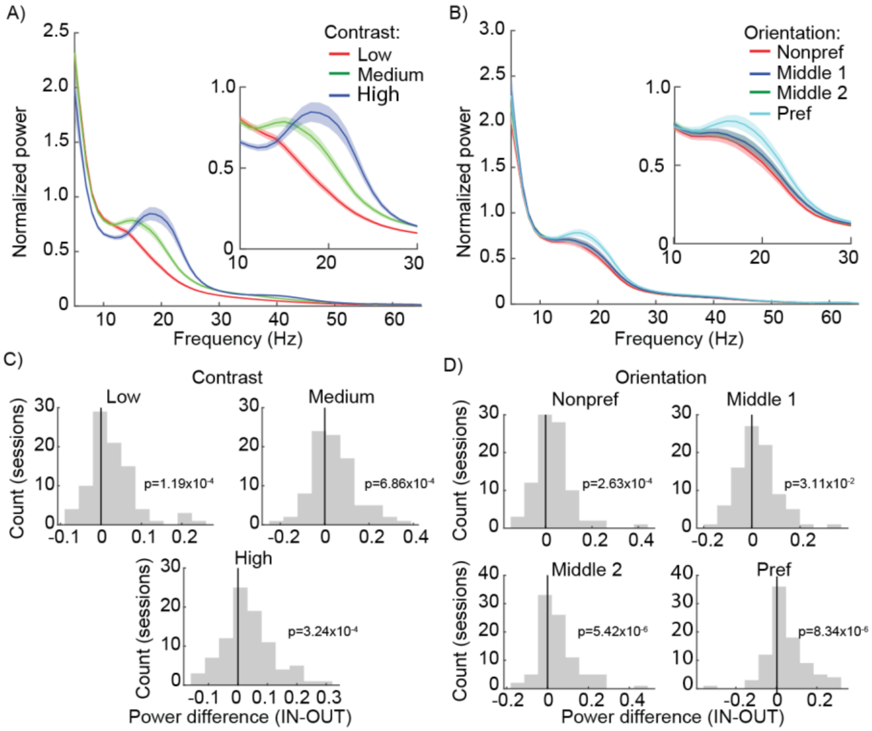

Figure S2) LFP αβ power and frequency reflect stimulus properties and content of WM. We examined how the top-down WM signal and bottom-up visual input altered the LFP power spectrum. A, B) Average LFP power spectrum during the delay (n = 88 sessions), for the memory IN condition, in response to backgrounds with three different contrasts (A) and different orientations (B, sorted according to the preferred orientation of neurons recorded at the same time as the LFP). More effective background stimuli (higher contrast, preferred orientation) stimuli were associated with a larger peak in the β range, with the frequency of peak β LFP power increasing from ~14 Hz for low contrast to ~19 Hz for high contrast stimuli. Inset shows 10-30Hz range. Shaded areas show standard error of mean (SEM). The β range LFP thus reflected the properties of the visual stimulus. C, D) Histograms show the distribution of LFP power differences (IN – OUT) in the αβ range (10-20 Hz) across sessions for each contrast (C, Contrast: $\Delta Power_{low}$=0.026±0.060, $\Delta Power_{middle}$=0.041±0.102, $\Delta Power_{high}$=0.036±0.086) or orientation (D, Orientation: $\Delta Power_{nonpref}$=0.032±0.080, $\Delta Power_{middle1}$=0.022±0.084, $\Delta Power_{middle2}$=0.049±0.094, $\Delta Power_{pref}$=0.048±0.104). αβ LFP power significantly increased in the IN condition for all background stimuli (p-values shown on plots; Wilcoxon signed-rank). Thus a WM signal directed toward the RF of the V4 LFP site significantly increased β power across all contrasts and orientations of background stimuli.

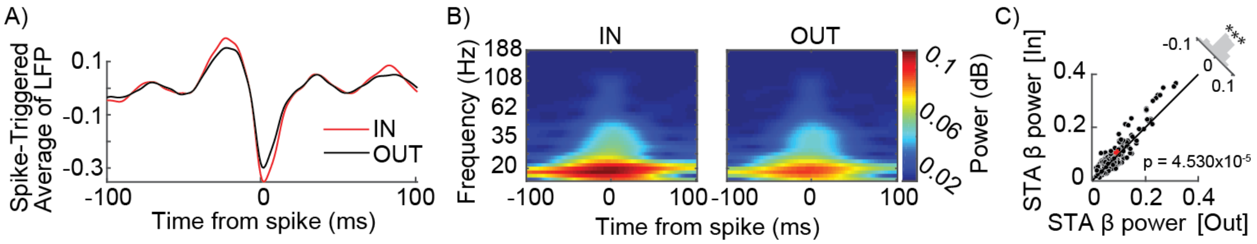

Figure S3) β power of the LFP STA is modulated by working memory. We calculated the spike-triggered average (STA) of the LFP during the delay period, and compared its spectral properties between memory conditions. A) STA of the normalized LFP during the delay period, for the population of 145 V4 neurons, for memory IN (red) and OUT (black) conditions. Notice that the peak and trough values are greater in the IN condition. B) Power of the LFP STA across frequency and time from the spike for memory IN (left) and OUT (right) conditions. The STA power is primarily in the lower frequencies. C) Scatter plot of STA power, averaged in β range, for IN versus OUT conditions across 88 sessions. Histogram shows the distribution of the difference in STA β power across neurons (OUT-IN). There was significantly greater β power in the STA for the IN condition (STA β $power_{IN}$=0.104±0.076, STA β $power_{OUT}$=0. 092±0.063, $p<10^{-4}$, Wilcoxon signed-rank).

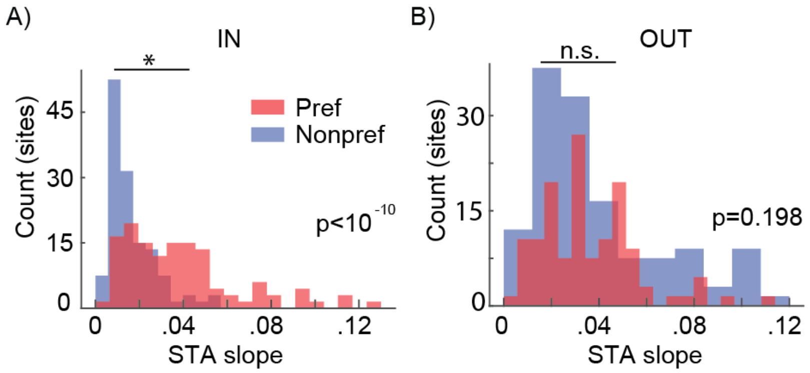

Figure S4) Spike-LFP relationship depends on stimulus orientation and WM condition. We tested how the slope of the LFP STA around the time of spikes varied based on background stimulus orientation and memory condition. A, B) Histogram of the distribution of slopes (abs (V_{peak} – V_{trough}) / ($time_{peak}$ – $time_{trough}$)) of the STA of the normalized LFP across neurons, for the preferred orientation (Pref, red) and nonpreferred orientation (Nonpref, blue) of the background stimulus (calculated based on neuron's firing rates during fixation), for the IN (A) and OUT (B) conditions. For the IN condition, the slope of the LFP STA was significantly different between the preferred and nonpreferred background stimuli (IN condition: $Slope_{Pref}$=0.039±0.003, $Slope_{Nonpref}$=0.016±0.002, $p<10^{-10}$, Wilcoxon signed-rank). For the OUT condition, there was no difference in the slope of the LFP STA based on the background stimulus (OUT condition: $Slope_{Pref}$=0.040±0.003, $Slope_{Nonpref}$=0.036±0.004, p=0.198, Wilcoxon signed-rank). This follows the pattern of results reported for changes in the STA slope for high vs. low contrast background stimuli in Figure 2D.

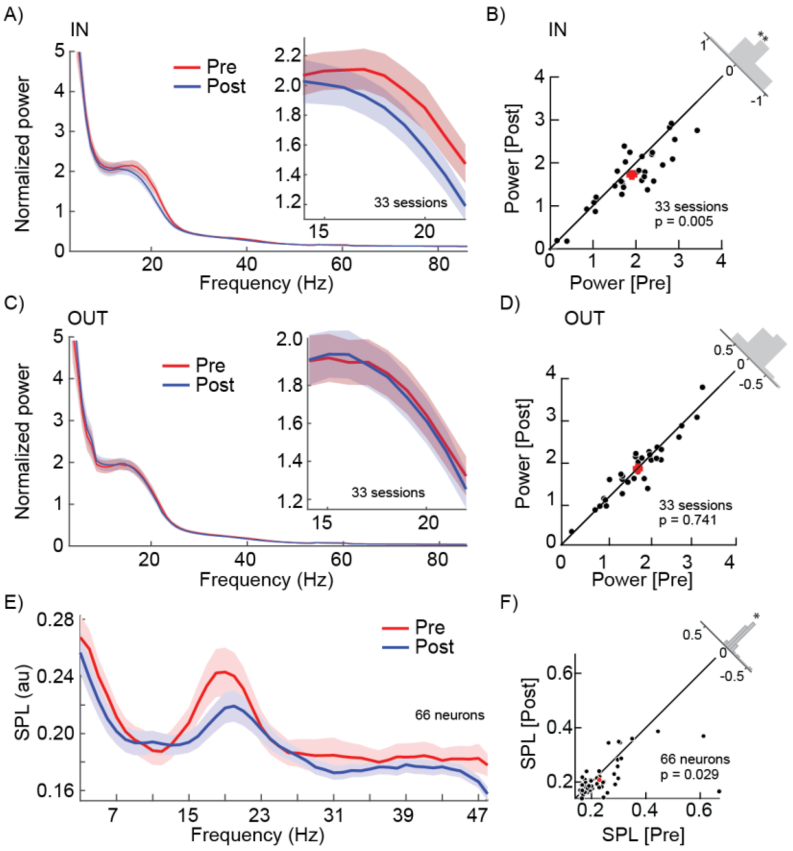

Figure S5) FEF inactivation reduces WM-driven β LFP power and SPL in V4. To test whether FEF activity was responsible for the WM-driven boost in V4 β LFP power and SPL, we compared V4 LFP power and the synchronization of spikes to the LFP oscillation at the same sites before vs. after pharmacological FEF inactivation. A) Normalized V4 LFP power spectrum during the delay period of the IN condition, before (Pre, red) and after (Post, blue) FEF inactivation. Inset shows power in β range (14-22 Hz). Shaded areas show standard error of mean (SEM). B) Scatter plot of V4 β LFP power for the IN condition, Pre versus Post FEF inactivation across 33 sessions. Histogram in upper right shows the distribution of the β power difference across sessions. There was significantly more β power for Pre inactivation condition (β $power_{Pre}$=1.907±0.228, β $power_{Post}$=1.720±0.296, p=0.005, Wilcoxon signed-rank). C, D) Same as A, B but for OUT condition. There was no change in V4 β LFP power before vs. after FEF inactivation for the OUT condition (β $power_{Pre}$=1.767±0.242, β $power_{Post}$=1.751±0.271, p=0.741, Wilcoxon signed-rank). E) Average SPL between V4 spikes and the phase of LFP oscillations as a function of LFP frequency during the delay period, for Pre (red) and Post (blue) FEF inactivation during the IN condition. Shaded areas show standard error of mean (SEM). F) Scatter plot of SPL in the β range for each neuron, Pre versus Post FEF inactivation. Histogram in the upper right shows the distribution of change in SPL (Post-Pre) across neurons. V4 β SPL was significantly lower following FEF inactivation (SPL_{pre}=0.227±0.093, SPL_{post}=0.202±0.054, p=0.005, Wilcoxon signed-rank). These results demonstrate that FEF activity is necessary for the WM-driven boost in V4 β LFP power and SPL observed when remembering a location in the shared RF.

Table S1: Parameter values utilized in the neural mass and neural field models. See Methods section for definitions of each parameter. For details on parameter selection and exploration of parameter space, see (Nesse et al., 2023).

Parameter	Value
V_t	-50 mV
V_r	-65 mV
V_l	-65 mV
C	1 mF
τ_i	16 ms
τ_e	5 ms
g	1/15 mS
σ_0	3 nA
I_{e0}	-1.71 nA
I_{i0}	-1.21 nA
Δ_{stim}	0.018 nA
Δ_{WM}	0.015 nA
Δ_{σ}	0.03 nA
w_{ee}	0.9
w_{ii}	1.9
w_{ie}	1
w_{ei}	2
κ	5.0625
κ_s	20
σ_z	*0.02*
τ_z	*50 ms*

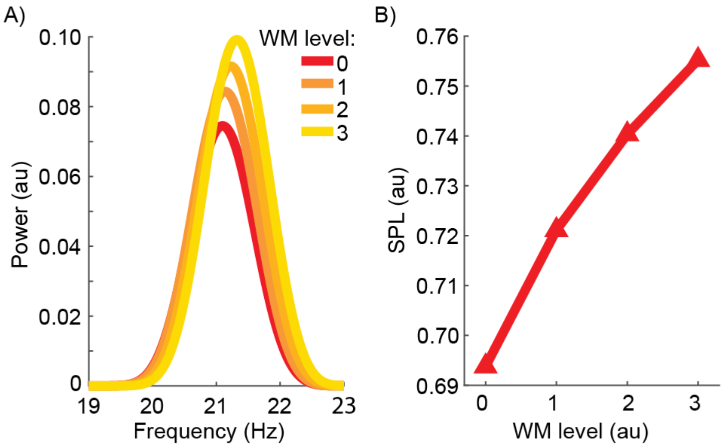

Figure S6) Neural field model replicates key experimental findings of WM's impact on LFP power and spike timing. We built a neural field model of visual areas receiving bottom-up sensory and top-down WM input signals (Nesse et al., 2023). This model exhibits β oscillations and locking of spikes to these oscillations; here we examine how oscillatory power and SPL change with increasing WM input strength, and compare to previously reported experimental results (where memory IN vs. OUT correspond to different WM input strengths). A) Model LFP power across frequencies, as a function of WM input strength (color). The peak LFP power and frequency of maximum power both increase with greater WM input. Increases in β LFP power with WM input have been experimentally observed in MT (Bahmani et al., 2018), and in V4 (Fig. 1I&J). B) Model SPL in the β band as a function of WM input strength. β SPL increases with greater WM input, as experimentally observed in V4 (Fig. 2B). Thus the model replicated key findings regarding the effect of WM on LFP oscillatory power and SPL in visual areas.

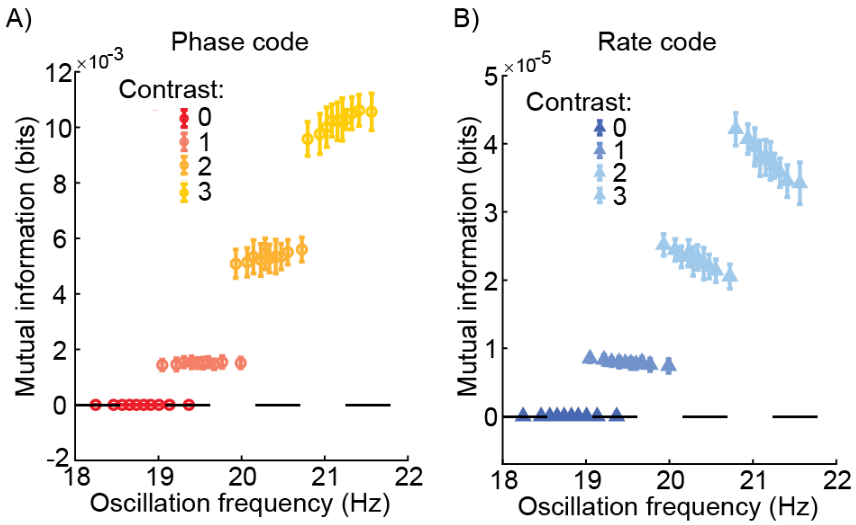

Figure S7) Model-derived mutual information for phase and rate codes. To test the relationship between information content and oscillation frequency in our model, we introduced noise into the strength of our WM input, and separated data for each oscillatory cycle with a given stimulus input strength based on oscillation frequency. We then calculated the mutual information between the two stimuli over four contrast levels, segmented by oscillation frequency deciles, using the phase code (A) or rate code (B). The results calculated using mutual information replicate the pattern of results reported using the information gain measure in figure 4E: phase coded information increased with increasing oscillation frequency while rate coded information decreased at higher oscillation frequencies.

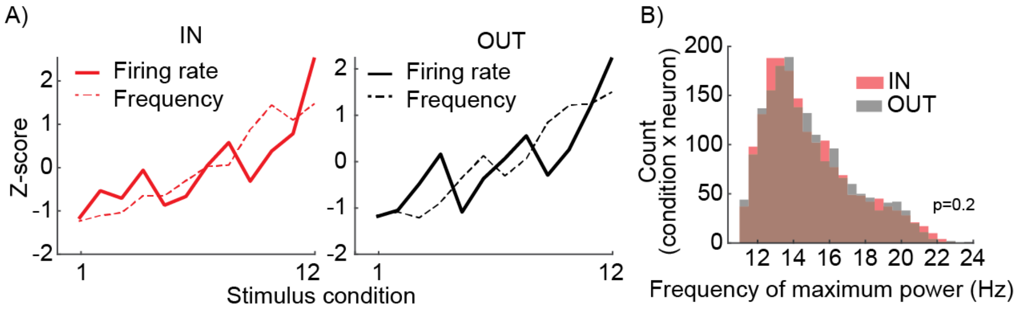

Figure S8) Firing rate and frequency of maximum power as a function of stimulus efficacy. In our experimental data, we looked for whether the observed relationship between oscillation frequency and firing rate varied for different combinations of background stimuli and memory condition. A) Changes in firing rate (solid line) and frequency of the maximum β LFP power (dashed line) as a function of background stimulus for memory IN (left) and OUT (right) conditions, during the delay period. Z-score reflects the difference from the average firing rate or average frequency of maximum power across all 12 stimulus conditions. The 12 background stimuli (orientation and contrast combinations) were sorted according to the firing rate of the neuron during the fixation period (weakest stimulus #1 to strongest stimulus #12). B) Histogram shows the distribution of peak LFP frequencies during the delay period, calculated separately for the 12 stimulus conditions for each neuron, for the memory IN (red) and OUT (grey) conditions ($PeakFreq_{IN}$=14.853±2.461, $PeakFreq_{OUT}$=14.885±2.427, p=0.210, Wilcoxon signed-rank). These results show that the relationship between LFP frequency and firing rate in V4 is consistent across different bottom-up inputs and memory conditions.

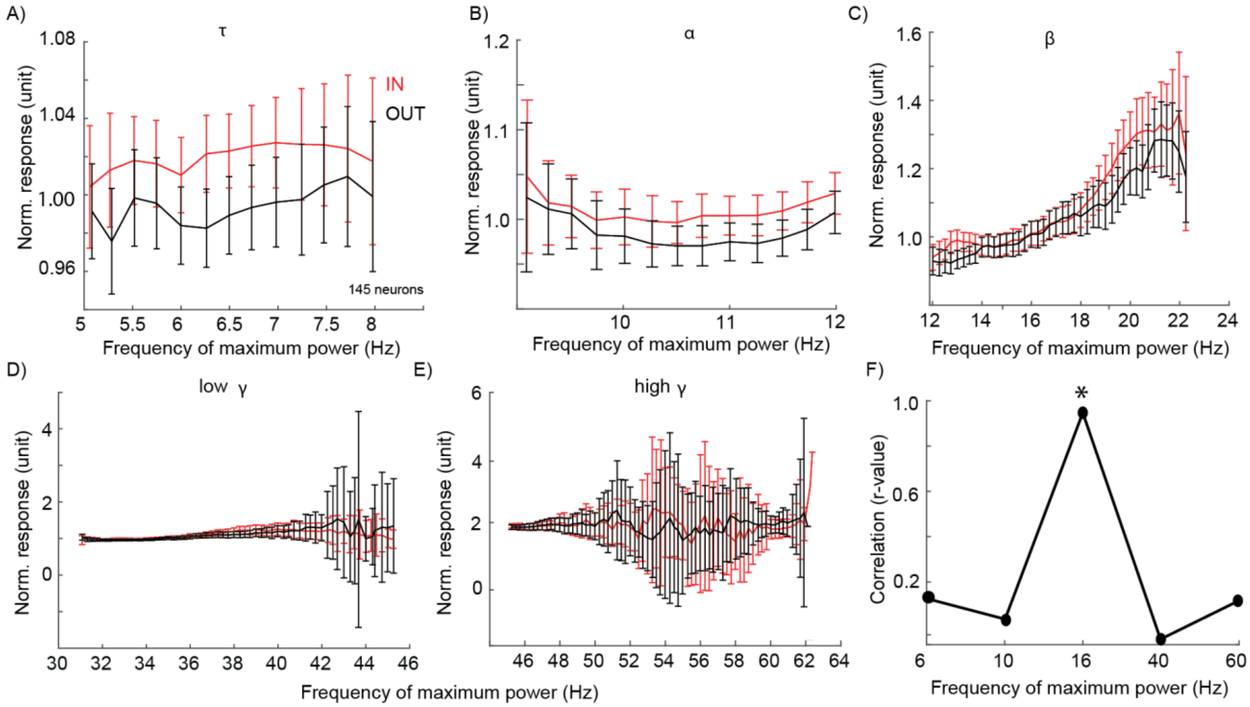

Figure S9) Correlation between firing rate and peak frequency is specific to β frequency oscillations. We examined multiple frequency bands to determine whether the relationship between the frequency of LFP oscillations and the firing rate of V4 neurons was general, or unique to the β band. A-E) The relationship between firing rate and the peak frequency during the delay period in different frequency bands: 4-8 Hz (theta, A), 8-12 Hz (alpha, B), 12-30 Hz (beta, C), 30-50 Hz (low gamma, D), 50-70 Hz (high gamma, E). As in figure 4G, we show average normalized response as a function of peak frequency, pooled across subsamples of trials from each of 145 V4 neurons (100 subsamples/neuron), during the IN (red) and OUT (blue) conditions. Plot shows mean±SE for all subsamples with the peak frequency indicated on the x-axis; actual subsample peak frequencies did not always span the full range of the band examined. F) The correlation between firing rate and frequency of maximum power is significant for β oscillations, but not for any other frequency bands. X-axis shows the frequency band, and y-axis is the r-value of the correlation between normalized firing rate and frequency of maximum power in that frequency band (Pearson correlation, $r_{\tau}=0.145$, $p_{\tau}=0.620$; $r_{\alpha}=0.060$, $p_{\alpha}=0.839$; $r_{\beta}=0.921$, $p_{\beta}<10^{-18}$; $r_{l\gamma}=-0.024$, $p_{l\gamma}=0.836$; $r_{h\gamma}=0.136$, $p_{h\gamma}=0.347$). These results show that the correlation between V4 LFP frequency and firing rate was unique to the β frequency range.
